# Association of Primary Tumor Resection with Survival in De Novo Stage IV Colorectal Cancer: A Retrospective Cohort Study with Propensity Score Matching

**DOI:** 10.3390/curroncol33080467

**Published:** 2026-08-05

**Authors:** Hatice Ayyıldız Sevim, Galip Can Uyar, Hayriye Şahinli

**Affiliations:** Department of Medical Oncology, Ankara Etlik City Hospital, Ankara 06170, Türkiye; g.can_uyar@hotmail.com (G.C.U.); dr.hayriye@hotmail.com (H.Ş.)

**Keywords:** colorectal cancer, de novo stage IV, primary tumor resection, progression-free survival, overall survival

## Abstract

Background: The role of primary tumor resection (PTR) in patients with de novo stage IV colorectal cancer remains controversial, particularly in the context of patient selection and tumor biology. This study aimed to evaluate the association between PTR and survival outcomes and to identify clinical, molecular, and inflammatory-nutritional prognostic factors in patients with de novo stage IV colorectal cancer. Methods: Medical records of 204 patients with de novo stage IV colorectal adenocarcinoma treated at Ankara Etlik City Hospital from December 2022 to December 2025 were reviewed retrospectively. Patients were grouped according to PTR status. Baseline clinicopathological features, molecular tumor profile, metastatic disease extent, treatment characteristics, and inflammatory-nutritional markers were recorded. Survival outcomes were assessed in terms of progression-free survival (PFS) and overall survival (OS) using the Kaplan–Meier method and Cox proportional hazards regression models, as well as 1:1 propensity score matching and sensitivity analyses. Results: PTR was performed in 114 patients (55.9%), while 90 patients (44.1%) did not undergo PTR. Patients who underwent PTR were younger, had better Eastern Cooperative Oncology Group (ECOG) performance status, and more frequently had single-organ metastatic disease. In the unmatched cohort, patients who underwent PTR had longer median PFS and OS than those without PTR (15.88 vs. 11.03 months and 16.14 vs. 11.54 months, respectively; both log-rank *p* < 0.001). In the adjusted Cox models, PTR corresponded to lower risks of progression (hazard ratio [HR]: 0.50; 95% confidence interval [CI]: 0.34–0.74; *p* < 0.001) and death (HR: 0.48; 95% CI: 0.30–0.77; *p* = 0.002), whereas BRAF mutation showed higher risks of progression (HR: 3.00; 95% CI: 1.70–5.29; *p* < 0.001) and death (HR: 3.42; 95% CI: 1.83–6.38; *p* < 0.001). In the propensity score–matched cohort comprising 50 matched pairs, PTR remained associated with a lower risk of progression or death (HR: 0.59; 95% CI: 0.40–0.87; *p* = 0.007), whereas its association with OS was not statistically significant (HR: 0.69; 95% CI: 0.42–1.13; *p* = 0.141). Sensitivity analyses generally yielded estimates favoring PTR, although the statistical significance of the association with OS varied across analyses. Patients with higher prognostic nutritional index (PNI) values showed more favorable survival outcomes. Conclusions: In this retrospective cohort, PTR was consistently associated with longer PFS, whereas its association with OS was less robust across the adjusted analyses. Because these patients had a more favorable baseline profile, these associations should be viewed with caution and in relation to patient selection. BRAF mutation and PNI emerged as important prognostic factors, supporting a multidimensional approach to survival assessment that incorporates metastatic disease burden, tumor biology, and inflammatory-nutritional status.

## Simple Summary

Colorectal cancer that has already spread to other organs is difficult to treat, and it is still unclear whether removing the original bowel tumor helps patients live longer. We reviewed 204 patients with stage IV colorectal cancer. Patients who had the original tumor removed had a longer time before disease progression, although the overall survival advantage was less certain after further adjustment. However, they were also younger, in better general health, and more often had cancer that had spread to only one organ. This means that the survival difference may not have been caused by surgery alone. A change in the BRAF gene was linked to shorter survival, while better nutritional and immune status was linked to longer survival. These findings suggest that surgery should not be routinely offered to every patient. Decisions should consider symptoms, overall health, how far the cancer has spread, and tumor characteristics. Further studies are needed to identify the patients most likely to benefit.

## 1. Introduction

Colorectal cancer remains one of the major contributors to cancer-related mortality worldwide [1]. Although the 5-year survival rate exceeds 90% when colorectal cancer is diagnosed at an early stage, it decreases to approximately 65% in patients with regional lymph node involvement and falls below 10% in those with distant metastatic disease [2]. Metastatic disease remains a major clinical challenge in colorectal cancer; a substantial proportion of patients present with distant metastases at diagnosis, and metastatic progression is also frequently observed during the disease course [3]. Despite substantial progress in treatment options over recent years, survival in stage IV colorectal cancer remains limited, and the overall prognosis continues to be poor [4].

Although systemic therapy remains the cornerstone of treatment in metastatic disease, management of the primary tumor continues to represent an important clinical challenge, particularly in patients presenting with metastatic disease at diagnosis. In this context, the role of primary tumor resection (PTR) is closely related to symptom status, metastatic disease extent, and the planned systemic treatment strategy [5,6]. While surgery is generally considered necessary in the presence of primary tumor-related complications such as bleeding, obstruction, or perforation, the survival contribution of PTR in asymptomatic patients with unresectable metastatic disease remains uncertain.

Randomized studies have evaluated whether PTR performed before systemic therapy provides a survival advantage over systemic therapy alone in patients with asymptomatic unresectable metastatic colorectal cancer. Overall, these trials have not demonstrated a clear improvement in overall survival with routine PTR performed before systemic therapy [7,8]. However, interpretation of the available evidence is limited by factors such as early trial termination and insufficient patient accrual in some studies. Moreover, recent meta-analytic data suggest that any potential benefit of PTR may be restricted to selected patients with a lower tumor burden who are able to receive more intensive systemic chemotherapy [9]. Thus, the subgroup of patients with stage IV colorectal cancer who may benefit from PTR remains unclear, highlighting the need for prognostic markers that can better guide patient selection.

Identifying patients with de novo stage IV colorectal cancer who are more likely to gain a survival advantage from PTR remains challenging. Therefore, treatment decisions should not be based solely on the presence of the primary tumor, but should also consider performance status, metastatic disease burden, tumor biology, inflammatory-nutritional status, and suitability for systemic therapy. This study examined the relationship between PTR and survival outcomes in patients with de novo stage IV colorectal cancer and assessed clinical, molecular, and inflammatory-nutritional factors that may influence prognosis.

## 2. Methods

### 2.1. Study Selection

In this retrospective study, patients diagnosed with de novo stage IV colorectal cancer at the Department of Medical Oncology, Ankara Etlik City Hospital, between December 2022 and December 2025 were evaluated. Patients with histopathologically confirmed colorectal adenocarcinoma and radiologically documented distant metastatic disease were included. Patients were excluded if they had multiple primary malignancies, synchronous second cancers, inflammatory bowel disease-associated colorectal cancer, incomplete key clinical or survival data, or insufficient follow-up information. The cohort was divided according to PTR status, and the groups were compared with respect to baseline clinicopathological features, treatment characteristics, and survival outcomes. The decision to perform primary tumor resection was not based on a single prespecified criterion. It was individualized following multidisciplinary assessment, taking into account the presence and severity of primary tumor-related symptoms, such as obstruction, bleeding, or perforation, as well as the patient’s performance status, metastatic disease burden and distribution, resectability, and the planned systemic treatment strategy. Patients did not receive identical systemic treatment; first-line chemotherapy regimens were generally similar between the groups, whereas the distribution of targeted agents differed. Ethical approval was obtained from the Scientific Research Evaluation and Ethics Committee of Ankara Etlik City Hospital (Decision No: AEŞH-BADEK1-2026-107; Date of approval: 18 February 2026).

### 2.2. Data Collection and Definitions

Patient records were reviewed to collect demographic data, ECOG performance status, primary tumor site, histological characteristics, molecular findings, number and sites of metastatic involvement, systemic treatments, and PTR status. Laboratory assessment included neutrophil, lymphocyte, and platelet counts, as well as albumin, CEA, CA19-9, LDH, CRP, hemoglobin, and creatinine levels; measurements closest to treatment initiation were used for the analyses.

The institutional laboratory reference ranges were 1.8–7.5 × 10^9^/L for neutrophils, 0.8–3.2 × 10^9^/L for lymphocytes, 150–450 × 10^9^/L for platelets, 35–52 g/L for albumin, <5 ng/mL for carcinoembryonic antigen, <37 U/mL for carbohydrate antigen 19-9, <223 U/L for lactate dehydrogenase, 0–5 mg/L for C-reactive protein, and 12–16 g/dL for hemoglobin in women and 13.5–17.5 g/dL in men.

Inflammatory and nutritional indices were calculated using these laboratory parameters. The systemic immune-inflammation index (SII) was calculated as platelet count × neutrophil count/lymphocyte count. The prognostic nutritional index (PNI) was calculated using the following formula: serum albumin level (g/L) + 5 × total lymphocyte count (10^9^/L). Because of their distributional characteristics, SII, CEA, and CA19-9 values were included in the analyses after logarithmic transformation.

### 2.3. Statistical Analysis

All statistical analyses were performed using IBM SPSS Statistics, version 25.0 (IBM Corp., Armonk, NY, USA), and Python, version 3.14 (Python Software Foundation, Wilmington, DE, USA). Python-based analyses were implemented using the pandas, NumPy, and statsmodels libraries for data management, variable transformation, propensity score procedures, and survival modeling. Before analysis, the dataset was checked for invalid dates, internally inconsistent survival intervals, and out-of-range values.

The distributions of continuous variables were evaluated using histograms, quantile–quantile plots, and the Kolmogorov–Smirnov and Shapiro–Wilk tests. Because the continuous variables generally showed non-normal distributions, they were reported as medians with interquartile ranges. Categorical variables were summarized as numbers and percentages. Baseline characteristics were compared between patients who underwent primary tumor resection and those who did not using the Mann–Whitney U test for continuous variables and the Pearson chi-square test or Fisher’s exact test for categorical variables, as appropriate.

Progression-free survival was defined as the interval from the diagnosis of de novo stage IV colorectal cancer to radiologically or clinically documented disease progression or death from any cause, whichever occurred first. Overall survival was measured from diagnosis to death from any cause. Patients without the relevant event were censored at the date of their last documented assessment. Survival distributions were estimated using the Kaplan–Meier method and compared using the two-sided log-rank test. The mean observed follow-up duration was summarized with its 95% confidence interval and range, whereas the median follow-up duration was estimated using the reverse Kaplan–Meier method.

Associations between candidate prognostic factors and survival outcomes were initially examined using univariable Cox proportional hazards regression. Multicollinearity among candidate covariates was assessed using variance inflation factors, with values below 5 considered acceptable. Variables with a univariable *p* value < 0.10 and no evidence of relevant multicollinearity were entered simultaneously into the outcome-specific multivariable Cox models. Effect estimates were reported as hazard ratios with 95% confidence intervals.

To reduce baseline treatment-selection bias, a propensity score for primary tumor resection was estimated using multivariable logistic regression. The propensity score model included pretreatment age, sex, Eastern Cooperative Oncology Group performance status, primary tumor location, histologic differentiation, KRAS and BRAF mutation status, number of metastatic organs, metastatic-site pattern, log-transformed systemic immune-inflammation index, prognostic nutritional index, log-transformed carcinoembryonic antigen, log-transformed carbohydrate antigen 19-9, and lactate dehydrogenase. First-line chemotherapy regimen and targeted-agent use were not included because their timing could have occurred after, or have been influenced by, primary tumor resection.

Patients were matched 1:1 without replacement using optimal cardinality matching within a caliper of 0.20 standard deviations of the logit of the propensity score. Fine-balance constraints were applied to the number of metastatic organs, metastatic-site pattern, NRAS status, microsatellite instability status, and HER2 category; additional balance constraints were applied to C-reactive protein, hemoglobin, and creatinine. Covariate balance was evaluated using absolute standardized mean differences. An absolute standardized mean difference below 0.10 was considered indicative of adequate balance. For categorical variables with more than two levels, balance was assessed using category-specific standardized mean differences, with the largest absolute value reported for the overall variable. *p* values were not used to assess post-matching balance.

Survival outcomes in the propensity score–matched cohort were evaluated using Kaplan–Meier estimates and Cox proportional hazards models. To account for the dependence introduced by matching, the Cox models used robust sandwich standard errors clustered by matched-pair identifier.

Because complete resection of metastatic disease could independently affect survival, a sensitivity analysis excluded patients who underwent R0 metastasectomy after primary tumor resection. After excluding these patients, the conventional multivariable Cox models were refitted in the restricted cohort. The propensity score was then re-estimated using the same pretreatment covariates, and 1:1 matching was repeated using the same caliper and cardinality-matching framework. Cox models in the re-matched restricted cohort again incorporated robust sandwich standard errors clustered by matched-pair identifier.

To address potential immortal-time bias arising from the interval between diagnosis and surgery, additional time-dependent Cox analyses were performed. Primary tumor resection was treated as a time-varying exposure: PTR(t) was coded as 0 from diagnosis until the date of surgery and as 1 thereafter. Patients who did not undergo primary tumor resection remained coded as 0 throughout follow-up. The data were structured in start–stop format, and robust sandwich standard errors were clustered at the patient level. Adjusted time-dependent models used the same outcome-specific baseline covariate sets as the corresponding conventional multivariable Cox models.

All statistical tests were two-sided, and *p* values < 0.05 were considered statistically significant. *p* values were reported to three decimal places, with values below 0.001 presented as *p* < 0.001.

## 3. Results

A total of 204 patients with de novo stage IV colorectal cancer were included in the study. The baseline demographic, clinical, molecular, and laboratory characteristics of the entire cohort are presented in Supplementary Table S1. The median age was 68.3 years (interquartile range [IQR]: 58.7–74.0), and 122 patients (59.8%) were aged ≥65 years. Most patients were male (59.8%), and the majority had an ECOG performance status of 0–1 (81.4%). Primary tumor resection was performed in 114 patients (55.9%), whereas 90 patients (44.1%) did not undergo PTR.

The primary tumor was most frequently located in the left colon (40.7%), followed by the right colon (32.8%) and rectum (26.5%). Single-organ metastasis was present in 142 patients (69.6%), and the most frequent metastatic pattern was liver-only metastasis (52.0%). Molecular evaluation revealed KRAS mutations in 104 patients (51.0%) and BRAF mutations in 15 patients (7.4%). Most patients received oxaliplatin-based chemotherapy as first-line treatment (89.2%), and anti-VEGF therapy was the most commonly used targeted agent (58.3%).

When patients were stratified by PTR status, those in the resection group were younger and had better ECOG performance status (*p* = 0.018 and *p* = 0.008, respectively). Primary tumor site, KRAS mutation status, metastatic extent, and metastatic pattern also differed between the groups. Single-organ metastasis was more common in the PTR group, whereas more extensive metastatic disease was more prominent among patients who did not undergo PTR. First-line chemotherapy regimens were similar; however, targeted treatment distribution differed significantly between the groups (*p* < 0.001). In addition, Log-CEA, Log-CA19-9, and LDH levels were significantly higher in patients without PTR (all *p* < 0.001). Other comparisons are presented in Table 1.

The surgical, pathological, and postoperative characteristics of patients who underwent PTR are presented in Supplementary Table S2. Among these patients, 57 (50.0%) had primary tumor-related symptoms, whereas 57 (50.0%) were asymptomatic. The most frequently performed surgical procedures were right hemicolectomy (33.3%) and low anterior resection (32.5%). Pathological evaluation showed pT3 disease in 50.9% of patients and pT4 disease in 43.0%. Regarding nodal involvement, pN1 disease was the most common category (43.0%). Postoperative surgical complications included intra-abdominal abscess, surgical site infection, and postoperative bleeding; surgery-related mortality occurred in 2 patients (1.8%).

The mean observed follow-up duration was 16.04 months (95% CI, 14.67–17.40 months; range, 0.82–58.09 months). The median follow-up duration estimated using the reverse Kaplan–Meier method was 21.85 months (95% CI, 19.78–25.79 months). During follow-up, 162 progression events and 108 deaths were observed. In the overall study population, median PFS was 13.02 months (IQR: 8.38–20.82), and median OS was 16.40 months (IQR: 10.58–23.92).

**Figure 1:**
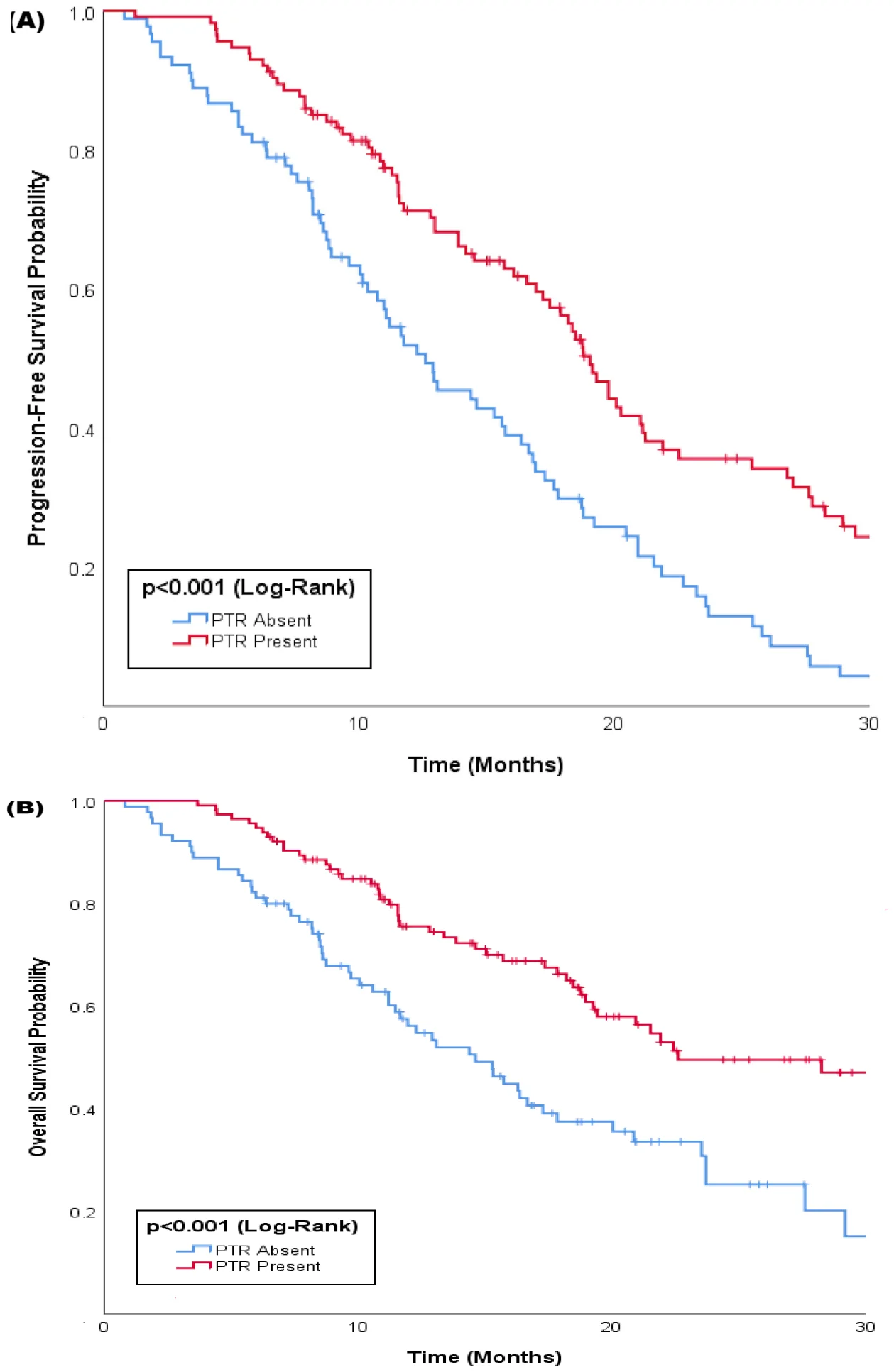
Kaplan–Meier curves stratified by primary tumor resection (PTR) status in the overall cohort. (**A**) Comparison of progression-free survival (PFS) between patients with and without PTR. (**B**) Comparison of overall survival (OS) between patients with and without PTR. Abbreviations: PTR, primary tumor resection; PFS, progression-free survival; OS, overall survival. *p*-values were obtained using the log-rank test; vertical tick marks indicate censored observations.

Kaplan–Meier curves stratified by PTR status demonstrated longer survival durations in the resection group. Median PFS was 11.03 months in patients without PTR and 15.88 months in those with PTR (log-rank *p* < 0.001; Figure 1A). Median OS was 11.54 and 16.14 months in the corresponding groups, respectively (log-rank *p* < 0.001; Figure 1B).

**Figure 2:**
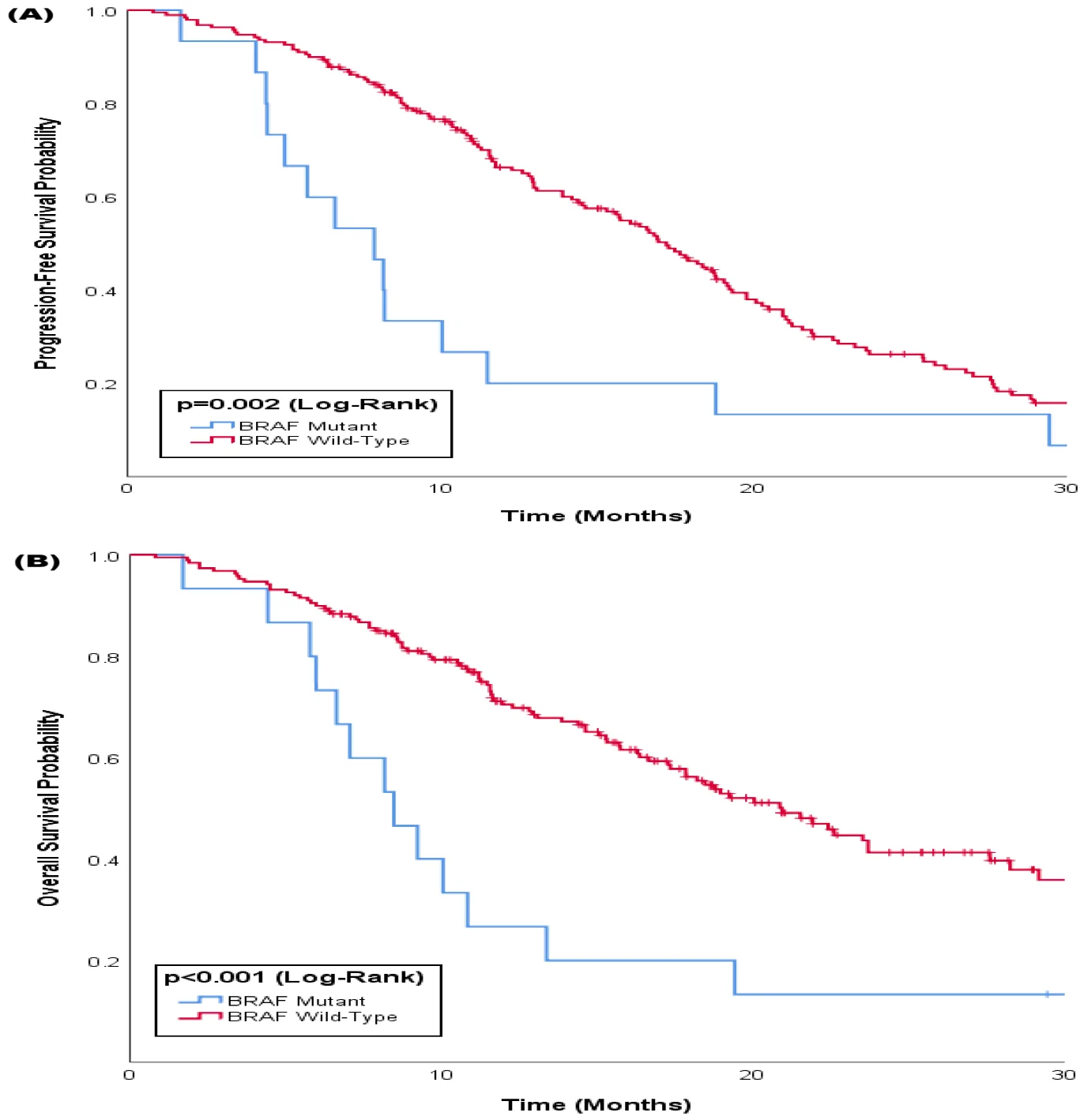
Kaplan–Meier curves stratified by BRAF mutation status in the overall cohort. (**A**) Comparison of progression-free survival (PFS) between BRAF-mutant and BRAF wild-type patients. (**B**) Comparison of overall survival (OS) between BRAF-mutant and BRAF wild-type patients. Abbreviations: BRAF, v-raf murine sarcoma viral oncogene homolog B1; PFS, progression-free survival; OS, overall survival. *p*-values were obtained using the log-rank test; vertical tick marks indicate censored observations.

Kaplan–Meier curves stratified by BRAF mutation status showed shorter survival durations in patients with BRAF-mutant disease. Median PFS was 7.88 months in the BRAF-mutant group and 17.21 months in the BRAF wild-type group (log-rank *p* = 0.002; Figure 2A). Median OS was 8.47 and 20.92 months in the corresponding groups, respectively (log-rank *p* < 0.001; Figure 2B).

In the univariable Cox analysis for PFS, ECOG PS ≥ 2, involvement of at least two metastatic organs, BRAF mutation, lower PNI, elevated Log-CEA, elevated Log-CA19-9, and no PTR were linked to shorter PFS. After adjustment in the multivariable model, BRAF mutation retained its independent prognostic effect and corresponded to a markedly higher risk of progression (HR: 3.00; 95% CI: 1.70–5.29; *p* < 0.001). By contrast, increasing PNI was related to a reduced risk of progression (HR: 0.96; 95% CI: 0.94–0.99; *p* = 0.007). Patients who underwent PTR also had a lower progression risk (HR: 0.50; 95% CI: 0.34–0.74; *p* < 0.001). The remaining variables did not show independent statistical significance in the adjusted model. Detailed Cox regression results are provided in Table 2.

In the univariable Cox analysis for OS, age ≥65 years, ECOG PS ≥ 2, involvement of at least two metastatic organs, BRAF mutation, lower PNI, elevated Log-CEA, elevated Log-CA19-9, and no PTR were linked to shorter OS. In the multivariable model, age ≥ 65 years was associated with a higher risk of death (HR: 1.63; 95% CI: 1.07–2.51; *p* = 0.025). BRAF mutation was also identified as an independent adverse prognostic factor for OS (HR: 3.42; 95% CI: 1.83–6.38; *p* < 0.001). By contrast, increasing PNI was related to a reduced risk of death (HR: 0.94; 95% CI: 0.92–0.97; *p* < 0.001). Patients who underwent PTR also had a lower mortality risk (HR: 0.48; 95% CI: 0.30–0.77; *p* = 0.002). Detailed Cox regression results are provided in Table 3.

Propensity score matching yielded 50 matched pairs, comprising 50 patients who underwent primary tumor resection and 50 patients who did not. After matching, the absolute standardized mean differences for all included pretreatment covariates were below 0.10. In particular, the absolute standardized mean difference decreased from 0.584 to 0.000 for the number of metastatic organs and from 0.530 to 0.000 for metastatic-site pattern. Covariate balance before and after matching is presented in Supplementary Table S3 and Supplementary Figure S1. In the matched cohort, primary tumor resection was associated with a lower risk of progression or death (HR, 0.59; 95% CI, 0.40–0.87; *p* = 0.007). Median progression-free survival was 17.2 months in the primary tumor resection group and 12.9 months in the no-resection group (log-rank *p* = 0.018). The association with overall survival favored primary tumor resection but was not statistically significant (HR, 0.69; 95% CI, 0.42–1.13; *p* = 0.141). Median overall survival was 19.0 and 15.3 months, respectively (log-rank *p* = 0.167). The corresponding Kaplan–Meier curves are shown in Supplementary Figure S2.

After excluding 49 patients who underwent R0 metastasectomy, primary tumor resection remained associated with progression-free survival in the restricted cohort of 155 patients (HR, 0.65; 95% CI, 0.43–0.98; *p* = 0.038), whereas the association with overall survival did not reach statistical significance (HR, 0.63; 95% CI, 0.39–1.02; *p* = 0.063). The propensity score was re-estimated within this restricted cohort, yielding 43 matched pairs. In the re-matched analysis, the progression-free survival estimate remained borderline in favor of primary tumor resection (HR, 0.64; 95% CI, 0.41–1.00; *p* = 0.050; exact *p* = 0.0496), while the association with overall survival remained non-significant (HR, 0.66; 95% CI, 0.39–1.11; *p* = 0.118).

In the time-dependent Cox analyses, in which primary tumor resection was modeled as a time-varying exposure, resection was associated with a lower risk of progression or death (HR, 0.67; 95% CI, 0.49–0.92; *p* = 0.014) and a lower risk of death (HR, 0.65; 95% CI, 0.42–0.99; *p* = 0.045). Detailed findings from the propensity score and sensitivity analyses are provided in Supplementary Table S4, and the primary and sensitivity estimates are summarized in Table 4.

## 4. Discussion

This study examined the relationship between PTR status and survival outcomes in patients with de novo stage IV colorectal cancer, while also assessing clinical, molecular, and inflammatory-nutritional variables that may contribute to prognosis. In the entire cohort, patients who underwent PTR showed longer PFS and OS in both Kaplan–Meier analyses and multivariable Cox models. However, patients who underwent PTR were younger, had better ECOG performance status, and more frequently had single-organ metastatic disease. After propensity score matching, the association remained statistically significant for PFS, whereas the association with OS was attenuated and was no longer statistically significant. Sensitivity analyses excluding patients who underwent R0 metastasectomy and time-dependent Cox analyses generally yielded estimates favoring PTR, although the statistical significance of the OS association varied across analyses. Therefore, the association between PTR and improved survival should not be attributed to surgery alone, but should be interpreted in light of patient selection, baseline clinical characteristics, and the retrospective design of the study.

In our cohort, the median PFS and OS were 15.88 and 16.14 months, respectively, in patients who underwent PTR, compared with 11.03 and 11.54 months in those who did not. These findings differ from randomized evidence that has not demonstrated a clear survival advantage for routine PTR performed before systemic therapy. In the JCOG1007/iPACS trial, median OS was 25.9 months in the PTR plus chemotherapy arm and 26.7 months in the chemotherapy-alone arm, with no significant difference between the groups [7]. Similarly, in the combined analysis of the SYNCHRONOUS and CCRe-IV trials, median OS was 16.7 months in the PTR arm and 18.6 months in the non-PTR arm, without a significant survival advantage in favor of PTR [8]. A recent meta-analysis similarly found that upfront PTR did not improve OS in asymptomatic patients with synchronous unresectable metastatic disease, although a possible benefit in cancer-specific survival was reported [10]. Although the median OS in our PTR group was numerically close to that reported for the PTR arm in the SYNCHRONOUS/CCRe-IV analysis, the association with OS was attenuated and was no longer statistically significant after propensity score matching. This result is more consistent with randomized evidence and suggests that the significant association observed in the entire cohort may have been partly influenced by differences in study design and patient selection.

Among molecular variables, BRAF mutation showed a clear adverse prognostic effect for both PFS and OS in our cohort. Median OS was 8.47 months in patients with BRAF-mutant disease, whereas it was 20.92 months in those with BRAF wild-type disease. This result is in line with previous studies reporting more aggressive tumor behavior and shorter survival in BRAF-mutant metastatic colorectal cancer [11]. In a real-world study from Alberta, Canada, median OS was reported as 8.21 months for patients with BRAF-mutant metastatic colorectal cancer and 20.03 months for those with BRAF wild-type disease [12]. These values closely mirror the survival durations observed in our cohort. BRAF V600E mutation has also been described in approximately 8–12% of metastatic colorectal cancers and has been linked to an unfavorable prognosis [13]. Given the small number of patients with BRAF-mutant disease, this finding should be interpreted cautiously; however, the clear reduction in both PFS and OS supports the adverse prognostic impact of BRAF mutation in de novo stage IV colorectal cancer.

In contrast to BRAF mutation, higher PNI values were related to more favorable PFS and OS in our study. This finding indicates that the patient’s inflammatory-nutritional status may influence prognosis in de novo stage IV colorectal cancer. Calculated from serum albumin level and peripheral lymphocyte count, PNI reflects both nutritional reserve and immune status. Systematic reviews and meta-analyses evaluating patients with colorectal cancer have reported poorer survival outcomes in those with low PNI [14,15]. Therefore, inflammatory-nutritional status may add prognostic information beyond tumor biology and metastatic disease extent.

In the multivariable Cox model for OS, age ≥65 years was related to an increased risk of death, whereas age did not show independent significance in the PFS analysis. This result is consistent with previous studies reporting the effect of older age on survival in metastatic colorectal cancer. Data from the ARCAD clinical trials program also showed an association between age and survival outcomes in patients with metastatic colorectal cancer [16]. In our cohort, ECOG performance status and the number of involved metastatic organs were related to survival in univariable analyses, but neither retained independent prognostic significance in the multivariable models. This suggests that prognosis in de novo stage IV colorectal cancer is not determined solely by performance status or metastatic disease extent.

Clinically, these findings indicate that PTR should not be regarded as a routine approach for all patients with de novo stage IV colorectal cancer, particularly because the association with OS was not consistent across all adjusted analyses. Recent meta-analytic data also indicate that the potential benefit of PTR may become more apparent in selected patients with a lower tumor burden who are able to receive more intensive systemic therapy [9]. Accordingly, the decision to perform PTR should be made within a comprehensive clinical assessment that considers disease extent, suitability for systemic therapy, and tumor biology, rather than the presence of the primary tumor alone [17].

This study has several limitations. Its retrospective nature prevents the complete exclusion of selection-related bias. In addition, the use of data from a single center may restrict the transferability of the results to other clinical settings. The follow-up duration was relatively short, which may have limited the ability to detect longer-term survival differences between the groups, particularly for OS. Baseline differences between patients who did and did not undergo PTR should also be taken into account when interpreting the survival findings. Nevertheless, this study offers valuable information for patient selection by considering surgical status together with clinical, molecular, and inflammatory-nutritional variables. Prospective multicenter studies are needed to better define the patient groups most likely to benefit from PTR.

## 5. Conclusions

In this retrospective cohort, primary tumor resection was consistently associated with longer progression-free survival, whereas its association with overall survival was less robust across the adjusted analyses. However, the more favorable baseline profile of the resection group indicates that these associations should not be interpreted as a direct surgical effect alone. BRAF mutation was linked to poorer survival, whereas higher prognostic nutritional index values were related to better outcomes. Therefore, in de novo stage IV colorectal cancer, the decision to perform primary tumor resection should be guided not only by the presence of the primary tumor, but also by metastatic disease extent, molecular tumor features, and the patient’s inflammatory-nutritional status.

## Figures and Tables

**Table 1:** Baseline Demographic, Clinical, Molecular, and Laboratory Characteristics According to Primary Tumor Resection (PTR) Status.

Characteristic	No PTR (*n* = 90, 44.1%)	PTR (*n* = 114, 55.9%)	*p*-Value
Age (Years), Median (IQR)	69.7 (59.7–76.4)	66.1 (54.1–71.3)	0.018
Sex, *n* (%)			0.290
Female	32 (35.6%)	50 (43.9%)	
Male	58 (64.4%)	64 (56.1%)	
ECOG Performance Status, *n* (%)			0.008
0–1	67 (74.4%)	99 (86.8%)	
≥2	23 (25.6%)	15 (13.2%)	
Primary Tumor Location, *n* (%)			0.030
Right Colon	21 (23.3%)	46 (40.4%)	
Left Colon	40 (44.4%)	43 (37.7%)	
Rectum	29 (32.2%)	25 (21.9%)	
Histology, *n* (%)			0.802
Well/Moderately Differentiated	64 (71.1%)	84 (73.7%)	
Poorly/Undifferentiated	26 (28.9%)	30 (26.3%)	
Molecular Profile, *n* (%)			
KRAS			0.032
Mutant	54 (60.0%)	50 (43.9%)	
Wild-Type	36 (40.0%)	64 (56.1%)	
NRAS			0.789
Mutant	3 (3.3%)	2 (1.8%)	
Wild-Type	87 (96.7%)	112 (98.2%)	
BRAF			0.949
Mutant	6 (6.7%)	9 (7.9%)	
Wild-Type	84 (93.3%)	105 (92.1%)	
MSI Status			1.000
MSS (Stable)	89 (98.9%)	112 (98.2%)	
MSI-H (High)	1 (1.1%)	2 (1.8%)	
HER2			0.539
Negative	27 (30.0%)	41 (36.0%)	
Positive	2 (2.2%)	4 (3.5%)	
Unknown/Not Tested	61 (67.8%)	69 (60.5%)	
Metastasis Status, *n* (%)			
Number of Metastatic Organs			<0.001
1 Organ	51 (56.7%)	91 (79.8%)	
2 Organs	38 (42.2%)	19 (16.7%)	
3 Organs	1 (1.1%)	4 (3.5%)	
Metastatic Sites			<0.001
Liver Only	42 (46.7%)	64 (56.1%)	
Lung Only	2 (2.2%)	5 (4.4%)	
Peritoneum Only	4 (4.4%)	14 (12.3%)	
Liver + Peritoneum	18 (20.0%)	4 (3.5%)	
Lung + Liver	17 (18.9%)	6 (5.3%)	
Other	7 (7.8%)	21 (18.4%)	
First-Line Treatment, *n* (%)			
Chemotherapy Regimen			0.152
Oxaliplatin-based	80 (88.9%)	102 (89.5%)	
Irinotecan-based	4 (4.4%)	1 (0.9%)	
5-FU-based	5 (5.6%)	5 (4.4%)	
Oxaliplatin + Irinotecan	1 (1.1%)	6 (5.3%)	
Targeted Agent			<0.001
Anti-VEGF	62 (68.9%)	57 (50.0%)	
Anti-EGFR	25 (27.8%)	19 (16.7%)	
None	3 (3.3%)	38 (33.3%)	
Log-SII	14.2 (13.7–14.4)	14.0 (13.4–14.4)	0.205
PNI	47.0 (42.5–51.7)	48.0 (44.3–52.7)	0.171
Log-CEA	4.7 (2.9–6.4)	2.2 (1.4–3.9)	<0.001
Log-CA19-9	4.4 (2.8–6.3)	3.4 (2.5–4.4)	<0.001
LDH (U/L)	305.0 (230.5–541.2)	214.0 (177.2–285.2)	<0.001
CRP (mg/L)	32.0 (11.0–65.5)	32.8 (9.2–63.8)	0.803
Hemoglobin (g/dL)	11.7 (10.3–13.1)	11.5 (10.2–12.6)	0.416
Creatinine (mg/dL)	0.8 (0.7–0.9)	0.8 (0.6–0.9)	0.146

Abbreviations: PTR: Primary Tumor Resection; ECOG: Eastern Cooperative Oncology Group; IQR: Interquartile Range; MSS: Microsatellite Stable; MSI-H: Microsatellite Instability-High; 5-FU: 5-Fluorouracil; VEGF: Vascular Endothelial Growth Factor; EGFR: Epidermal Growth Factor Receptor; SII: Systemic Immune-Inflammation Index; PNI: Prognostic Nutritional Index; CEA: Carcinoembryonic Antigen; CA19-9: Carbohydrate Antigen 19-9; LDH: Lactate Dehydrogenase; CRP: C-Reactive Protein KRAS: Kirsten rat sarcoma viral oncogene homolog; NRAS: neuroblastoma RAS viral oncogene homolog; BRAF: v-raf murine sarcoma viral oncogene homolog B1; HER2: human epidermal growth factor receptor 2.

**Table 2:** Cox Regression Analysis for Progression-Free Survival.

Variables	Univariable HR (95% CI)	Univariable *p*-Value	Multivariable HR (95% CI)	Multivariable *p*-Value
Age (≥65 vs. <65 years)	1.27 (0.92–1.75)	0.144	-	-
Sex (Male vs. Female)	0.86 (0.63–1.18)	0.357	-	-
ECOG PS (≥2 vs. 0–1)	2.07 (1.40–3.04)	<0.001	1.39 (0.90–2.13)	0.138
Primary Tumor Location (Right vs. Left/Rectum)	1.20 (0.86–1.66)	0.289	-	-
Metastatic Organs (≥2 vs. 1)	1.53 (1.08–2.16)	0.016	1.34 (0.94–1.93)	0.109
KRAS (Mutant vs. Wild-Type)	1.08 (0.79–1.48)	0.610	-	-
BRAF (Mutant vs. Wild-Type)	2.26 (1.32–3.86)	0.003	3.00 (1.70–5.29)	<0.001
Log-SII (Continuous)	1.18 (0.97–1.44)	0.096	1.00 (0.78–1.29)	0.389
PNI (Continuous)	0.96 (0.94–0.98)	<0.001	0.96 (0.94–0.99)	0.007
Log-CEA (Continuous)	1.10 (1.03–1.18)	0.005	0.97 (0.88–1.06)	0.482
Log-CA19-9 (Continuous)	1.15 (1.07–1.24)	<0.001	1.07 (0.97–1.17)	0.164
Primary Tumor Resection (Present vs. Absent)	0.50 (0.36–0.68)	<0.001	0.50 (0.34–0.74)	<0.001

Abbreviations: HR: Hazard Ratio; CI: Confidence Interval; ECOG PS: Eastern Cooperative Oncology Group Performance Status; SII: Systemic Immune-Inflammation Index; PNI: Prognostic Nutritional Index; CEA: Carcinoembryonic Antigen; CA19-9: Carbohydrate Antigen 19-9; KRAS, Kirsten rat sarcoma viral oncogene homolog; BRAF, v-raf murine sarcoma viral oncogene homolog B1. Variables demonstrating a *p*-value < 0.10 in the univariable analysis were included simultaneously in the multivariable Cox proportional hazards model (Enter method). Significant *p*-values (<0.05) are indicated in bold. HRs for continuous variables represent the change in risk per 1-unit increase in the respective index.

**Table 3:** Cox Regression Analysis for Overall Survival.

Variables	Univariable HR (95% CI)	Univariable *p*-Value	Multivariable HR (95% CI)	Multivariable *p*-Value
Age (≥65 vs. <65 years)	1.68 (1.12–2.52)	0.012	1.63 (1.07–2.51)	0.025
Sex (Male vs. Female)	1.02 (0.70–1.50)	0.912	-	-
ECOG PS (≥2 vs. 0–1)	2.76 (1.81–4.21)	<0.001	1.47 (0.91–2.38)	0.114
Primary Tumor Location (Right vs. Left/Rectum)	1.35 (0.91–2.00)	0.138	-	-
Metastatic Organs (≥2 vs. 1)	1.91 (1.29–2.84)	0.001	1.43 (0.93–2.18)	0.100
KRAS (Mutant vs. Wild-Type)	1.34 (0.91–1.97)	0.138	-	-
BRAF (Mutant vs. Wild-Type)	2.73 (1.52–4.89)	0.001	3.42 (1.83–6.38)	<0.001
Log-SII (Continuous)	1.19 (0.94–1.52)	0.153	-	-
PNI (Continuous)	0.94 (0.92–0.96)	<0.001	0.94 (0.92–0.97)	<0.001
Log-CEA (Continuous)	1.13 (1.05–1.23)	0.002	0.99 (0.88–1.10)	0.828
Log-CA19-9 (Continuous)	1.20 (1.09–1.31)	<0.001	1.07 (0.97–1.18)	0.183
Primary Tumor Resection (Present vs. Absent)	0.46 (0.31–0.68)	<0.001	0.48 (0.30–0.77)	0.002

Abbreviations: HR: Hazard Ratio; CI: Confidence Interval; ECOG PS: Eastern Cooperative Oncology Group Performance Status; SII: Systemic Immune-Inflammation Index; PNI: Prognostic Nutritional Index; CEA: Carcinoembryonic Antigen; CA19-9: Carbohydrate Antigen 19-9; KRAS, Kirsten rat sarcoma viral oncogene homolog; BRAF, v-raf murine sarcoma viral oncogene homolog B1. Variables demonstrating a *p*-value < 0.10 in the univariable analysis were included simultaneously in the multivariable Cox proportional hazards model (Enter method). Significant *p*-values (<0.05) are indicated in bold. HRs for continuous variables represent the change in risk per 1-unit increase in the respective index. Log-SII was not included in the multivariable OS model as its univariable *p*-value (0.153) exceeded the pre-defined 0.10 threshold.

**Table 4:** Association of primary tumor resection with progression-free and overall survival across the primary and sensitivity analyses.

Analysis	Population, *n*	PFS Events, *n*	PFS HR (95% CI)	*p* Value	Deaths, *n*	OS HR (95% CI)	*p* Value
Entire cohort, unadjusted Cox model	204	162	0.50 (0.36–0.68)	<0.001	108	0.46 (0.31–0.68)	<0.001
Entire cohort, multivariable-adjusted Cox model	204	162	0.50 (0.34–0.74)	<0.001	108	0.48 (0.30–0.77)	0.002
1:1 propensity score–matched cohort	100	85	0.59 (0.40–0.87)	0.007	57	0.69 (0.42–1.13)	0.141
R0 metastasectomy-excluded cohort, adjusted Cox model	155	129	0.65 (0.43–0.98)	0.038	94	0.63 (0.39–1.02)	0.063
R0 metastasectomy-excluded, re-matched cohort	86	75	0.64 (0.41–1.00)	0.050 *	53	0.66 (0.39–1.11)	0.118
Adjusted time-dependent Cox model	204	162	0.67 (0.49–0.92)	0.014	108	0.65 (0.42–0.99)	0.045

Abbreviations: CI, confidence interval; ECOG, Eastern Cooperative Oncology Group; HR, hazard ratio; OS, overall survival; PFS, progression-free survival; PSM, propensity score matching; PTR, primary tumor resection; R0, microscopically margin-negative metastasectomy. Hazard ratios below 1.00 favor PTR. The unadjusted models included PTR as the sole explanatory variable. The primary multivariable PFS model was adjusted for ECOG performance status, number of metastatic organs, BRAF mutation status, log-transformed systemic immune-inflammation index, prognostic nutritional index, log-transformed carcinoembryonic antigen, and log-transformed carbohydrate antigen 19-9. The primary multivariable OS model was adjusted for age category, ECOG performance status, number of metastatic organs, BRAF mutation status, prognostic nutritional index, log-transformed carcinoembryonic antigen, and log-transformed carbohydrate antigen 19-9. The propensity score–matched analysis included 50 matched pairs and used Cox proportional hazards models with robust sandwich standard errors clustered by matched-pair identifier. For the R0 metastasectomy-excluded sensitivity analysis, 49 patients who underwent R0 metastasectomy were excluded, and the propensity score was re-estimated within the remaining cohort before repeating 1:1 matching. In the time-dependent Cox analyses, PTR was modeled as a time-varying exposure, with PTR(t) = 0 from diagnosis until the date of surgery and PTR(t) = 1 thereafter; patient-clustered robust sandwich standard errors were used. Event counts represent the total number of progression or death events in the corresponding analysis population. All tests were 2-sided. * The exact *p* value for the R0 metastasectomy-excluded re-matched PFS analysis was 0.0496 and is presented as 0.050 after rounding to 3 decimal places.

## Data Availability

The datasets generated and/or analyzed during the current study are not publicly available due to institutional and patient privacy regulations but are available from the corresponding author upon reasonable request.

## Supplementary Materials

The following supporting information can be downloaded at: https://www.mdpi.com/article/10.3390/curroncol33080467/s1, Supplementary Table S1: Baseline demographic, clinical, molecular, and laboratory characteristics of the entire cohort; Supplementary Table S2: Surgical, pathological, and postoperative characteristics of patients who underwent primary tumor resection; Supplementary Table S3: Patient characteristics according to primary tumor resection status before and after 1:1 propensity score matching; Supplementary Table S4: Detailed propensity score and sensitivity analyses for progression-free and overall survival; Supplementary Figure S1: Covariate balance before and after 1:1 propensity score matching; Supplementary Figure S2: Kaplan–Meier survival estimates according to primary tumor resection status in the propensity score–matched cohort.
