# Supplementary material for: Association of Primary Tumor Resection with Survival in De Novo Stage IV Colorectal Cancer: A Retrospective Cohort Study with Propensity Score Matching
Source: Curr Oncol. 2026 Aug 5;33(8):467. doi: 10.3390/curroncol33080467 (PMC13510421; doi:10.3390/curroncol33080467)
Supplement: Supplementary file 1 [file curroncol-33-00467-s001.zip › curroncol-4417778-supplementary.pdf]

Supplementary Table S1: Baseline Demographic, Clinical, Molecular, and Laboratory Characteristics of the Entire Cohort

Characteristic	Entire Cohort (n = 204)
Age (Years), Median (IQR)	68.3 (58.7- 74.0)
Age Category, n (%)	
<65 years	82 (40.2%)
≥ 65 years	122 (59.8%)
Sex, n (%)	
Female	82 (40.2%)
Male	122 (59.8%)
ECOG Performance Status, n (%)	
0- 1	166 (81.4%)
≥ 2	38 (18.6%)
Primary Tumor Resection (PTR), n (%)	
Absent	90 (44.1%)
Present	114 (55.9%)
Primary Tumor Location, n (%)	
Right Colon	67 (32.8%)
Left Colon	83 (40.7%)
Rectum	54 (26.5%)
Histology, n (%)	
Well/Moderately Differentiated	148 (72.5%)
Poorly/Undifferentiated	56 (27.5%)
Molecular Profile, n (%)	
KRAS	
Mutant	104 (51.0%)
Wild-Type	100 (49.0%)
NRAS	
Mutant	5 (2.5%)
Wild-Type	199 (97.5%)
BRAF	
Mutant	15 (7.4%)
Wild-Type	189 (92.6%)
MSI Status	
MSS (Stable)	201 (98.5%)
MSI-H (High)	3 (1.5%)
HER2	
Negative	68 (33.3%)
Positive	6 (2.9%)
Unknown/Not Tested	130 (63.7%)
Metastasis Status, n (%)	
Number of Metastatic Organs	
1 Organ	142 (69.6%)
2 Organs	57 (27.9%)
3 Organs	5 (2.5%)
Metastatic Sites	
Liver Only	106 (52.0%)
Lung Only	7 (3.4%)
Peritoneum Only	18 (8.8%)
Liver + Peritoneum	22 (10.8%)
Lung + Liver	23 (11.3%)
Other	28 (13.7%)
First-Line Treatment, n (%)	
Chemotherapy Regimen	
Oxaliplatin-based	182 (89.2%)
Irinotecan-based	5 (2.5%)
5-FU-based	10 (4.9%)
Oxaliplatin + Irinotecan	7 (3.4%)
Targeted Agent	
Anti-VEGF	119 (58.3%)
Anti-EGFR	44 (21.6%)
None	41 (20.1%)
Laboratory & Inflammatory Indices, Median (IQR)	
Log-SII	14.1 (13.5- 14.4)
PNI	47.6 (43.6- 52.1)
Log-CEA	3.2 (1.7- 5.4)
Log-CA19-9	3.7 (2.6- 5.3)
LDH (U/L)	244.5 (192.8- 414.2)
CRP (mg/L)	32.8 (10.0- 64.5)
Hemoglobin (g/dL)	11.5 (10.3- 12.7)
Creatinine (mg/dL)	0.8 (0.6- 0.9)

Abbreviations: PTR: Primary Tumor Resection; IQR: Interquartile Range; ECOG: Eastern Cooperative Oncology Group; KRAS: Kirsten rat sarcoma viral oncogene homolog; NRAS: neuroblastoma RAS viral oncogene homolog; BRAF: v-raf murine sarcoma viral oncogene homolog

B1; MSS: Microsatellite Stable; MSI-H: Microsatellite Instability-High; 5-FU: 5-Fluorouracil; VEGF: Vascular Endothelial Growth Factor; EGFR: Epidermal Growth Factor Receptor; SII: Systemic Immune-Inflammation Index; PNI: Prognostic Nutritional Index; CEA: Carcinoembryonic Antigen; CA19-9: Carbohydrate Antigen 19-9; LDH: Lactate Dehydrogenase; CRP: C-Reactive Protein; HER2: human epidermal growth factor receptor 2.

Continuous variables are presented as medians with interquartile ranges (IQR). Categorical variables are presented as frequencies and valid percentages.

Supplementary Table S2: Surgical, Pathological, and Postoperative Characteristics of Patients Who Underwent Primary Tumor Resection

Surgical / Pathological Feature	Frequency (n)	Percentage (%)
Primary Tumor-Related Symptoms		
* Symptomatic (Yes)	57	50.0%
* Asymptomatic (No)	57	50.0%
Neoadjuvant Chemotherapy prior to PTR		
* Yes	41	36.0%
* No	73	64.0%
Post-PTR Chemotherapy		
* Yes	105	92.1%
* No	9	7.9%
Surgical Procedure Type		
* Ileocecal Resection	3	2.6%
* Right Hemicolectomy	38	33.3%
* Partial Colectomy	1	0.9%
* Left Hemicolectomy	13	11.4%
* Anterior Resection	8	7.0%
* Low Anterior Resection	37	32.5%
* Abdominoperineal Resection (APR)	3	2.6%
* Subtotal Colectomy	11	9.6%
Pathological T (pT) Stage		
* T0	3	2.6%
* T2	4	3.5%
* T3	58	50.9%
* T4	49	43.0%
Pathological N (pN) Stage		
* N0	31	27.2%
* N1	49	43.0%
* N2	32	28.1%
* N3	2	1.8%
Postoperative Surgical Complications		
* None	106	93.0%
* Intra-abdominal Abscess	5	4.4%
* Surgical Site Infection	2	1.8%
* Postoperative Bleeding	1	0.9%
Postoperative Morbidity (Clavien-Dindo Grade)		
* Grade 1	96	84.2%
* Grade 2	14	12.3%
* Grade 3	2	1.8%
* Grade 5 (Mortality)	2	1.8%

Abbreviations: PTR: Primary Tumor Resection; R0: Microscopically margin-negative resection; pT: Pathological Tumor size/invasion; pN: Pathological Node involvement; APR: Abdominoperineal Resection.

This table exclusively presents the data for the 114 patients who underwent primary tumor resection (PTR). Frequencies and valid percentages are shown.

Supplementary Table S3. Patient characteristics according to primary tumor resection status before and after 1:1 propensity score matching

Characteristic	Before matching: No PTR (n=90)	Before matching: PTR (n=114)	Absolute SMD	After matching: No PTR (n=50)	After matching: PTR (n=50)	Absolute SMD
Age, median (IQR), years	69.7 (59.7–76.4)	66.1 (54.1–71.3)	0.320	67.2 (56.5–71.3)	68.5 (59.8–73.9)	0.051
Sex, n (%)			0.170			0.041
Female	32 (35.6%)	50 (43.9%)		19 (38.0%)	20 (40.0%)	
Male	58 (64.4%)	64 (56.1%)		31 (62.0%)	30 (60.0%)	
ECOG performance status, n (%)			0.318			0.045
0–1	67 (74.4%)	99 (86.8%)		37 (74.0%)	36 (72.0%)	
≥2	23 (25.6%)	15 (13.2%)		13 (26.0%)	14 (28.0%)	

Characteristic	Before matching: No PTR (n=90)	Before matching: PTR (n=114)	Absolute SMD	After matching: No PTR (n=50)	After matching: PTR (n=50)	Absolute SMD
Primary tumor location, n (%)			0.372			0.043
Right colon	21 (23.3%)	46 (40.4%)		15 (30.0%)	15 (30.0%)	
Left colon	40 (44.4%)	43 (37.7%)		19 (38.0%)	18 (36.0%)	
Rectum	29 (32.2%)	25 (21.9%)		16 (32.0%)	17 (34.0%)	
Histology, n (%)			0.058			0.046
Well/moderately differentiated	64 (71.1%)	84 (73.7%)		38 (76.0%)	37 (74.0%)	
Poorly/undifferentiated	26 (28.9%)	30 (26.3%)		12 (24.0%)	13 (26.0%)	
KRAS status, n (%)			0.327			0.000
Mutant	54 (60.0%)	50 (43.9%)		26 (52.0%)	26 (52.0%)	
Wild-type	36 (40.0%)	64 (56.1%)		24 (48.0%)	24 (48.0%)	
BRAF status, n (%)			0.047			0.000
Mutant	6 (6.7%)	9 (7.9%)		3 (6.0%)	3 (6.0%)	
Wild-type	84 (93.3%)	105 (92.1%)		47 (94.0%)	47 (94.0%)	
Number of metastatic organs, n (%)			0.584			0.000
1 organ	51 (56.7%)	91 (79.8%)		38 (76.0%)	38 (76.0%)	
2 organs	38 (42.2%)	19 (16.7%)		11 (22.0%)	11 (22.0%)	
3 organs	1 (1.1%)	4 (3.5%)		1 (2.0%)	1 (2.0%)	
Metastatic site pattern, n (%)			0.530			0.000
Liver only	42 (46.7%)	64 (56.1%)		30 (60.0%)	30 (60.0%)	
Lung only	2 (2.2%)	5 (4.4%)		2 (4.0%)	2 (4.0%)	
Peritoneum only	4 (4.4%)	14 (12.3%)		4 (8.0%)	4 (8.0%)	
Liver + peritoneum	18 (20.0%)	4 (3.5%)		4 (8.0%)	4 (8.0%)	
Lung + liver	17 (18.9%)	6 (5.3%)		4 (8.0%)	4 (8.0%)	
Other	7 (7.8%)	21 (18.4%)		6 (12.0%)	6 (12.0%)	
Log-SII, median (IQR)	14.2 (13.7–14.4)	14.0 (13.4–14.4)	0.195	14.2 (13.4–14.4)	14.2 (13.3–14.6)	0.013
PNI, median (IQR)	47.0 (42.5–51.7)	48.0 (44.3–52.7)	0.175	47.5 (42.8–51.7)	47.0 (43.0–51.4)	0.056
Log-CEA, median (IQR)	4.7 (2.9–6.4)	2.2 (1.4–3.9)	1.019	3.8 (2.1–5.4)	3.6 (2.2–5.3)	0.075
Log-CA19-9, median (IQR)	4.4 (2.8–6.3)	3.4 (2.5–4.4)	0.557	4.0 (2.5–5.8)	3.6 (3.0–5.1)	0.027
LDH, median (IQR), U/L	305.0 (230.5–541.2)	214.0 (177.2–285.2)	0.407	282.0 (216.8–453.5)	261.0 (196.2–508.5)	0.018
CRP, median (IQR), mg/L	32.0 (11.0–65.5)	32.8 (9.2–63.8)	0.050	30.5 (10.0–67.0)	41.1 (9.9–69.4)	0.067
Hemoglobin, median (IQR), g/dL	11.7 (10.3–13.1)	11.5 (10.2–12.6)	0.108	11.5 (10.3–12.6)	11.4 (10.2–12.8)	0.068
Creatinine, median (IQR), mg/dL	0.8 (0.7–0.9)	0.8 (0.6–0.9)	0.062	0.80 (0.69–1.00)	0.80 (0.66–1.02)	0.027

Abbreviations: 5-FU, 5-fluorouracil; BRAF, B-Raf proto-oncogene; CA19-9, carbohydrate antigen 19-9; CEA, carcinoembryonic antigen; CRP, C-reactive protein; ECOG, Eastern Cooperative Oncology Group; EGFR, epidermal growth factor receptor; HER2, human epidermal growth factor receptor 2; IQR, interquartile range; KRAS, Kirsten rat sarcoma viral oncogene homolog; LDH, lactate dehydrogenase; MSI-H, microsatellite instability–high; MSS, microsatellite stable; NRAS, neuroblastoma RAS viral oncogene homolog; PNI, prognostic nutritional index; PTR, primary tumor resection; SII, systemic immune-inflammation index; SMD, standardized mean difference; VEGF, vascular endothelial growth factor.

Values are presented as median (interquartile range) or n (%), unless otherwise indicated. Propensity scores for undergoing primary tumor resection were estimated using a multivariable logistic regression model that included pretreatment age, sex, Eastern Cooperative Oncology Group performance status, primary tumor location, histologic differentiation, KRAS and BRAF mutation status, number and pattern of metastatic sites, log-transformed systemic immune-inflammation index, prognostic nutritional index, log-transformed carcinoembryonic antigen, log-transformed carbohydrate antigen 19-9, and lactate dehydrogenase. Patients were matched 1:1 without replacement using cardinality matching constrained by a caliper of 0.20 standard deviations of the logit of the propensity score, resulting in 50 matched pairs. Fine-balance constraints were applied to the number and pattern of metastatic sites and to NRAS, microsatellite instability, and HER2 categories; additional balance constraints were applied to C-reactive protein, hemoglobin, and creatinine. Covariate balance was assessed using absolute standardized mean differences. For continuous variables, standardized mean differences were calculated using the difference in means divided by the pooled standard deviation, although descriptive values are presented as medians and interquartile ranges because of non-normal distributions. For categorical variables with more than two levels, the parent row presents the largest absolute category-specific standardized mean difference. An absolute standardized mean difference <0.10 was considered indicative of adequate balance. P values were not reported for comparisons before or after matching because standardized mean differences provide a sample-size-independent assessment of covariate balance. First-line chemotherapy regimen and targeted-agent use were not included in the propensity score model or matching constraints

because their timing could have occurred after, or have been influenced by, primary tumor resection; these variables are presented for descriptive purposes only.

Supplementary Table S4. Detailed propensity score and sensitivity analyses for progression-free and overall survival

Panel A. Propensity score matching specifications and matched-cohort survival analyses

Specification	Description
Propensity score covariates	Age, sex, ECOG performance status, primary tumor location, histologic differentiation, KRAS and BRAF mutation status, number and pattern of metastatic sites, log-SII, PNI, log-CEA, log-CA19-9, and LDH
Matching algorithm	1:1 optimal cardinality matching without replacement
Caliper	0.20 standard deviations of the logit of the propensity score
Matched sample	50 matched pairs; 100 patients (50 PTR and 50 no PTR)
Unmatched patients	64 PTR and 40 no PTR patients
Covariate balance	All prespecified pretreatment covariates had absolute SMDs <0.10
Variance estimation	Matched-pair–clustered robust sandwich standard errors

Analysis	Population, n	PTR/No PTR, n	Events, PTR/No PTR, n	PTR HR (95% CI)	P value	Statistical approach
Matched-cohort PFS	100	50/50	40/45	0.59 (0.40–0.87)	0.007	Cox model with matched-pair–clustered robust standard errors
Matched-cohort OS	100	50/50	28/29	0.69 (0.42–1.13)	0.141	Cox model with matched-pair–clustered robust standard errors

Panel B. Sensitivity analyses excluding patients who underwent R0 metastasectomy

Specification	Description
Restricted cohort	Forty-nine patients who underwent post-PTR R0 metastasectomy were excluded; 155 patients remained, including 65 PTR patients without R0 metastasectomy and 90 no-PTR patients
Re-estimated propensity score	The propensity score was re-estimated within the restricted cohort using the same pretreatment covariates, caliper, and cardinality-matching framework
Restricted matched sample	43 matched pairs; 86 patients (43 PTR and 43 no PTR)
Unmatched patients	22 PTR and 47 no-PTR patients
Post-matching balance	Maximum absolute SMD, 0.092; all prespecified covariates had absolute SMDs <0.10

Analysis	Population, n	PTR/No PTR, n	Events, PTR/No PTR, n	PTR HR (95% CI)	P value	Statistical approach
R0-excluded adjusted PFS	155	65/90	50/79	0.65 (0.43–0.98)	0.038	Multivariable Cox proportional hazards model
R0-excluded adjusted OS	155	65/90	36/58	0.63 (0.39–1.02)	0.063	Multivariable Cox proportional hazards model
R0-excluded re-matched PFS	86	43/43	35/40	0.64 (0.41–1.00)	0.050*	Cox model with matched-pair–clustered robust standard errors
R0-excluded re-matched OS	86	43/43	25/28	0.66 (0.39–1.11)	0.118	Cox model with matched-pair–clustered robust standard errors

Panel C. Time-dependent Cox analyses addressing immortal-time bias

Specification	Description
Date-quality assessment	All 204 patients had valid diagnosis and endpoint dates, and all 114 patients who underwent PTR had a valid surgery date. No surgery date preceded the diagnosis date or occurred after the corresponding PFS or OS endpoint
Time-varying exposure definition	PTR(t) was coded as 0 from diagnosis until the date of surgery and as 1 thereafter. Patients who did not undergo PTR remained unexposed throughout follow-up
Counting-process data structure	The 204 patients contributed 259 start–stop intervals. Fifty-five patients underwent PTR after diagnosis and therefore contributed 2 intervals; 59 patients underwent PTR on the diagnosis date
Variance estimation	Patient-clustered robust sandwich standard errors

Analysis	Patients, n	Intervals, n	Events, n	Time-dependent PTR HR (95% CI)	P value	Adjustment
PFS, unadjusted	204	259	162	0.61 (0.45–0.83)	0.002	PTR(t) only
PFS, adjusted	204	259	162	0.67 (0.49–0.92)	0.014	Primary PFS covariate set
OS, unadjusted	204	259	108	0.59 (0.41–0.86)	0.006	PTR(t) only
OS, adjusted	204	259	108	0.65 (0.42–0.99)	0.045	Primary OS covariate set

Abbreviations: BRAF, B-Raf proto-oncogene; CA19-9, carbohydrate antigen 19-9; CEA, carcinoembryonic antigen; CI, confidence interval; ECOG, Eastern Cooperative Oncology Group; HR, hazard ratio; KRAS, Kirsten rat sarcoma viral oncogene homolog; LDH, lactate dehydrogenase; OS, overall survival; PFS, progression-free survival; PNI, prognostic nutritional index; PS, propensity score; PSM, propensity score matching; PTR, primary tumor resection; R0, microscopically margin-negative metastasectomy; SE, standard error; SII, systemic immune-inflammation index; SMD, standardized mean difference.

Event counts in Panels A and B are presented as PTR/no-PTR counts. Hazard ratios below 1.00 favor PTR. Propensity scores were estimated using a multivariable logistic regression model that included pretreatment age, sex, ECOG performance status, primary tumor location, histologic differentiation, KRAS and BRAF mutation status, number and pattern of metastatic sites, log-transformed SII, PNI, log-transformed CEA, log-transformed CA19-9, and LDH. Patients were matched 1:1 without replacement using optimal cardinality matching constrained by a caliper of 0.20 standard deviations of the logit of the propensity score. Covariate balance was assessed using absolute SMDs, with values <0.10 considered indicative of adequate balance. Matched analyses used Cox proportional hazards models with robust sandwich standard errors clustered by matched-pair identifier. For the R0-excluded sensitivity analysis, the propensity score was re-estimated after excluding 49 patients who underwent R0 metastasectomy. The R0-excluded multivariable PFS model was adjusted for ECOG performance status, number of metastatic organs, BRAF mutation status, log-SII, PNI, log-CEA, and log-CA19-9. The corresponding OS model was adjusted for age category, ECOG performance status, number of metastatic organs, BRAF mutation status, PNI, log-CEA, and log-CA19-9. In the time-dependent analyses, PTR was modeled as a time-varying exposure, with PTR(t)=0 before surgery and PTR(t)=1 from the date of surgery onward. The adjusted time-dependent models used the same outcome-specific covariate sets as the corresponding conventional multivariable models and incorporated patient-clustered robust sandwich standard errors. All tests were 2-sided. *The exact P value for the R0-excluded re-matched PFS analysis was 0.0496 and is displayed as 0.050 after rounding to 3 decimal places.

Supplementary Figure S1. Covariate balance before and after 1:1 propensity score matching

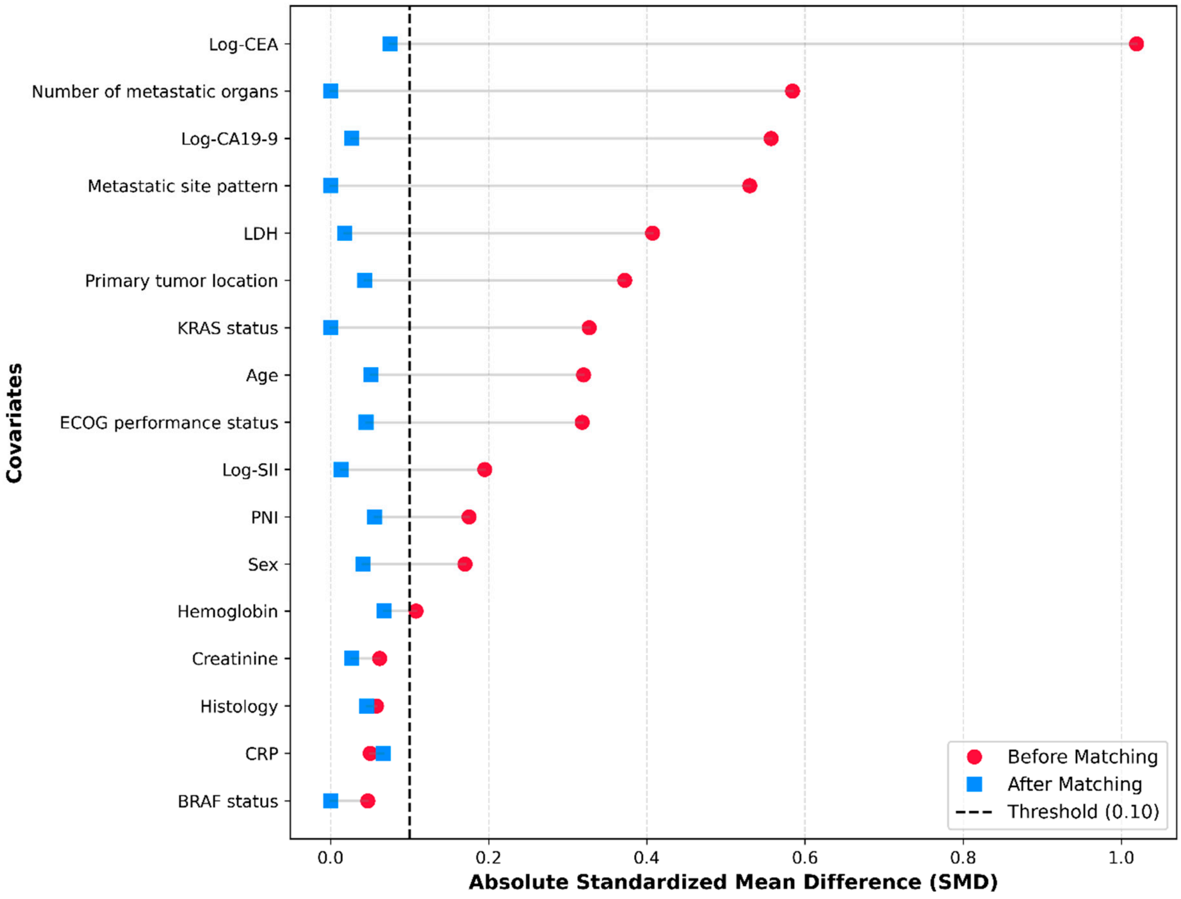

Abbreviations: BRAF, B-Raf proto-oncogene; CA19-9, carbohydrate antigen 19-9; CEA, carcinoembryonic antigen; CRP, C-reactive protein; ECOG, Eastern Cooperative Oncology Group; KRAS, Kirsten rat sarcoma viral oncogene homolog; LDH, lactate dehydrogenase; PNI, prognostic nutritional index; PSM, propensity score matching; SII, systemic immune-inflammation index; SMD, standardized mean difference.

The Love plot presents the absolute standardized mean differences for pretreatment covariates before and after propensity score matching. Red circles represent covariate imbalance before matching, blue squares represent covariate imbalance after matching, and the horizontal connecting lines illustrate the change in balance achieved through matching. Propensity scores for undergoing primary tumor resection were estimated using a multivariable logistic regression model that included age, sex, ECOG performance status, primary tumor location, histologic differentiation, KRAS and BRAF mutation status, number and pattern of metastatic sites, log-transformed SII, PNI, log-transformed CEA, log-transformed CA19-9, and LDH. Patients were matched 1:1 without replacement using optimal cardinality matching constrained by a caliper of 0.20 standard deviations of the logit of the propensity score, resulting in 50 matched pairs. Additional balance constraints were applied to CRP, hemoglobin, and creatinine. For continuous variables, SMDs were calculated as the difference in group means divided by the pooled standard deviation. For categorical variables with more than 2 levels, the plotted value represents the largest absolute category-specific SMD. The dashed vertical line indicates the prespecified threshold of 0.10 for acceptable covariate balance. All displayed covariates had an absolute SMD below 0.10 after matching.

Supplementary Figure S2. Kaplan–Meier survival estimates according to primary tumor resection status in the propensity score–matched cohort

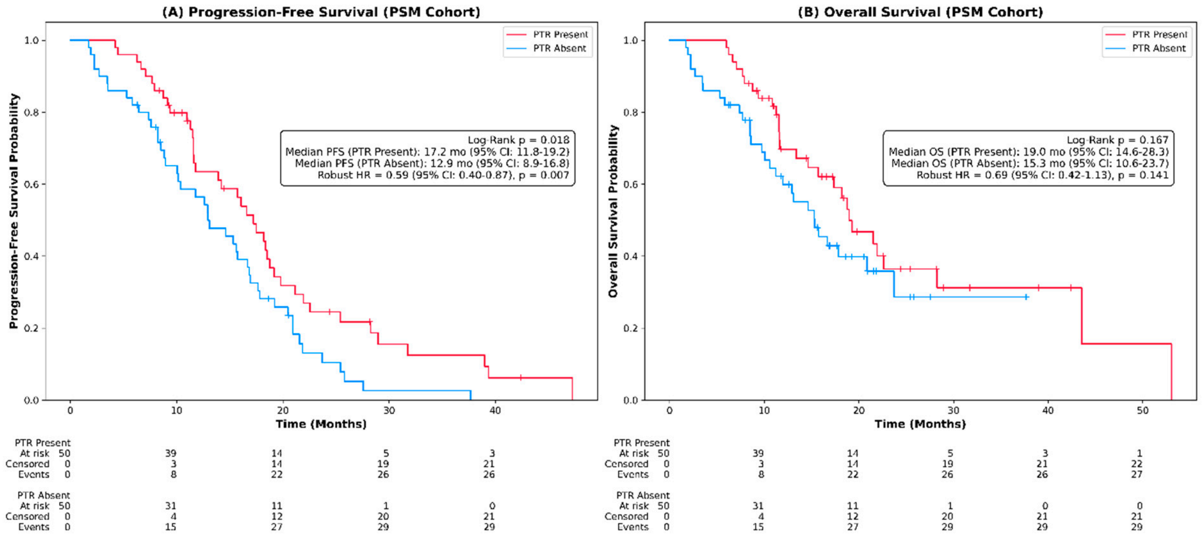

Abbreviations: CI, confidence interval; HR, hazard ratio; OS, overall survival; PFS, progression-free survival; PSM, propensity score matching; PTR, primary tumor resection.

Kaplan–Meier estimates of (A) progression-free survival and (B) overall survival according to PTR status in the 1:1 propensity score–matched cohort comprising 50 patients who underwent PTR and 50 matched patients who did not. Median PFS was 17.2 months (95% CI, 11.8–19.2 months) in the PTR group and 12.9 months (95% CI, 8.9–16.8 months) in the no-PTR group (log-rank P=0.018); in the matched-pair–clustered Cox model, PTR was associated with a lower risk of progression or death (HR, 0.59; 95% CI, 0.40–0.87; P=0.007). Median OS was 19.0 months (95% CI, 14.6–28.3 months) in the PTR group and 15.3 months (95% CI, 10.6–23.7 months) in the no-PTR group (log-rank P=0.167); the corresponding association with OS was not statistically significant (HR, 0.69; 95% CI, 0.42–1.13; P=0.141). Vertical tick marks indicate censored observations. The tables below each panel display the numbers at risk, cumulative censored observations, and cumulative events at the corresponding time points. Hazard ratios and 95% confidence intervals were estimated using Cox proportional hazards models with robust sandwich standard errors clustered by matched-pair identifier; HRs below 1.00 favor PTR.
